# Maternal Obesity and the Early Origins of Childhood Obesity: Weighing Up the Benefits and Costs of Maternal Weight Loss in the Periconceptional Period for the Offspring

**DOI:** 10.1155/2011/585749

**Published:** 2011-12-07

**Authors:** Song Zhang, Leewen Rattanatray, Janna L. Morrison, Lisa M. Nicholas, Shervi Lie, I. Caroline McMillen

**Affiliations:** Sansom Institute for Health Research, School of Pharmacy and Medical Sciences, University of South Australia, Adelaide, SA 5001, Australia

## Abstract

There is a need to understand the separate or interdependent contributions of maternal prepregnancy BMI, gestational weight gain, glycaemic control, and macronutrient intake on the metabolic outcomes for the offspring. Experimental studies highlight that there may be separate influences of maternal obesity during the periconceptional period and late gestation on the adiposity of the offspring. While a period of dietary restriction in obese mothers may ablate the programming of obesity, it is associated with an activation of the stress axis in the offspring. Thus, maternal obesity may result in epigenetic changes which predict the need for efficient fat storage in postnatal life, while maternal weight loss may lead to epigenetic changes which predict later adversity. Thus, development of dietary interventions for obese mothers during the periconceptional period requires a greater evidence base which allows the effective weighing up of the metabolic benefits and costs for the offspring.

## 1. Introduction

As the prevalence of obesity has increased in the developed world, more women are entering pregnancy with a high body mass index (BMI) in the overweight (BMI > 25 kg/m^2^) or obese (BMI > 30 kg/m^2^) range. As discussed in this paper, clinical and population studies have shown that maternal obesity results in fertility problems and suboptimal outcomes for the mother and her fetus during and after pregnancy [1]. It is also the case that maternal obesity is associated with an increase in BMI in the offspring during infancy, childhood, and later life. A recent scientific statement from the American Heart Association Council on Epidemiology and Prevention included a summary of the evidence for the prenatal determinants of obesity and highlighted that “*obesity among girls and women of childbearing age is producing a concomitant increase in rates of gestational diabetes which in turn is likely to lead to more obesity in the next generation. This vicious cycle may well fuel the obesity epidemic for decades to come*” [2]. This places a focus on the nutritional health of women before pregnancy and on the development of optimal weight loss interventions in overweight or obese women in the preconceptional period to improve pregnancy outcomes. This paper highlights recent evidence that suggests that the periconceptional period may represent a critical window during which exposure of the oocyte and/or embryo can independently contribute to an increased risk of obesity in the offspring. Further, this paper summarises evidence from experimental studies of the potential metabolic benefits and costs of weight loss during the periconceptional period in overweight and obese mothers for the offspring.

## 2. Maternal Prepregnancy BMI, Gestational Weight Gain, and Pregnancy Outcomes

During the past decade, the obesity epidemic in the developed world has resulted in an increase in the number of women entering pregnancy with a high body mass index (BMI) in the overweight (BMI > 25 kg/m^2^) or obese (BMI > 30 kg/m^2^) range. In the US, UK, and Australia, the prevalence of obesity in women aged between 20 and 39 years is around 28%, 20%, and 15%, respectively, as determined in studies carried out between 2000 and 2009 [1–5]. In the US, La Coursiere and colleagues [6] found that the incidence of women being either overweight or obese at the start of pregnancy increased from 25% to 35% between 1991 and 2001, and that the incidence of maternal obesity at delivery rose from 29% to 39% across the same period [6]. Although the proportion of women of reproductive age in the US who are overweight or obese appears to have plateaued recently, the proportion that is severely obese (BMI ≥ 40 kg/m^2^) is increasing. Similarly, two recent reports from different states in Australia found that the prevalence of maternal overweight and obesity was 34% in a population giving birth between 1998 and 2002 and 43% in a population measured at their first antenatal visit between 2001 and 2005 [7, 8]. Obese women are at increased risk of a range of pregnancy complications including gestational hypertension, preeclampsia, gestational diabetes mellitus (GDM), delivery of a large-for-gestational-age (LGA) infant, and an increase in Caesarean section rate [9]. 

In addition to the focus on the impact of maternal prepregnancy BMI on pregnancy outcomes, there has been a range of observational studies which suggest that there is also an association between gestational weight gain with short- and longer-term maternal outcomes [10]. The Institute of Medicine (IOM) in the US has recently revised its 1990 guidelines for gestational weight gain in underweight, normal weight, overweight, and obese women [11]. The new guidelines were developed based on a systematic evidence-based review [12] which found that the evidence that gestational weight gain was related to maternal pregnancy and postpartum outcomes was weak. There was, however, a moderate association between gestational weight gain and both Caesarean section delivery and maternal postpartum weight retention [12]. Some recent observational studies in obese women report better pregnancy outcomes at lower or negative gestational weight gains than in the revised IOM guidelines [10], but there are concerns that striving to achieve a lower gestational weight gain than that recommended in the new guidelines may have adverse outcomes [1]. The recent International Life Sciences Institute (ILSI) Europe Workshop on obesity in pregnancy concluded that given that the new IOM guidelines were based on observational studies, there was a pressing requirement for large randomised control trials which are adequately powered to test the validity of the recommendations [1]. Currently, it appears that for obese women, prepregnancy BMI is more associated with an increased risk of preeclampsia, GDM, and the delivery of an LGA infant than is gestational weight gain [9]. This places a focus on the nutritional health of women in the preconceptional period and on what weight loss interventions can be safely introduced in the obese woman seeking to become pregnant.

## 3. Maternal Prepregnancy BMI, Gestational Weight Gain, and Childhood Obesity

While it is not unexpected that the maternal nutritional and hormonal environment would determine fetal nutrient supply, fetal growth, and the body composition of the infant, the effects of the nutritional environment experienced *in utero* persist beyond fetal life. There is a U-shaped relationship between birth weight, and adult fat mass, with a higher prevalence of adult obesity occurring in individuals with birth weights at either the low or high end of the birth weight distribution [13–16]. A study in a large British cohort found that babies who were in the heaviest quintile of birth weight tended to have a high BMI in adult life independent of gender and that this relationship was largely accounted for by maternal weight [15]. A recent Danish study of 300,000 children also reported a remarkably stable association between having a birth weight greater than 4000 g and being overweight at 6–13 years of age in both girls and boys [17]. A retrospective US study of 8494 low-income families found that children (both boys and girls) born to obese mothers were twice as likely to be obese by 2 years of age [16]. There was a greater than 2-fold increase in the prevalence of obesity observed in children of obese mothers compared to those with mothers whose BMI was in the normal range.

Recent studies have investigated how a high maternal prepregnancy BMI, poor maternal glycaemic control, and total gestational weight gain may each contribute to a range of outcomes including an LGA baby and child obesity. In one report, siblings born to women who had undergone bariatric surgery for the treatment of severe obesity had a lower BMI and obesity risk than their siblings who were born prior to maternal surgery and weight loss [18]. In this latter study, it was not possible, due to the sample size, to determine whether there was any difference in the impact of a reduction in maternal weight on the metabolic outcomes in the male or female offspring. The findings of this latter study suggest that exposure to a high maternal BMI before and during pregnancy has important consequences for the metabolic health of the offspring. While some observational studies have found that there is an impact of gestational weight gain on outcomes including child obesity, the effect of gestational weight gain, independent of maternal prepregnancy BMI on offspring obesity, is not clear [10]. 

A high prepregnancy BMI is also associated with an increased risk of poor glucose tolerance and GDM, and recent randomised control trials provide evidence that there is a causal relationship between maternal glucose intolerance and the delivery of a macrosomic infant [19, 20]. A study on Pima Indians reported that there was an increased risk of obesity in offspring (male and female) born after the mother was diagnosed with diabetes compared with their siblings who were born before the mothers were diagnosed with diabetes [21]. Thus, exposure to an increase in high maternal glucose and insulin concentrations from conception results in a larger and fatter infant who is at increased risk of obesity in later life. Interestingly in a recent follow-up study of children whose mothers participated in a randomised control trial, treatment of mild GDM resulted in a reduction in macrosomia at birth but not in the BMI of offspring at 4-5 years of age [22]. While this null effect may be a consequence of the early age at which the children were studied, it is also possible that there are separate effects of exposure to GDM on fetal body growth and on the risk of increased adiposity in childhood. In this context, it is of note that maternal pre pregnancy weight and the associated level of maternal insulin resistance are strongly correlated with an infant's fat mass at birth, whereas the level of maternal insulin resistance in later pregnancy is correlated with birth weight and an infant's “fat-free” mass [23]. Thus, better glycaemic control in later pregnancy may have a greater impact on infant body growth than on the propensity for childhood obesity. Similarly, other studies have shown that there appear to be independent contributions of maternal pre pregnancy weight and maternal glucose intolerance during pregnancy to birth weight and the risk of adolescent obesity [24].

Finally, the role of maternal macronutrient and energy intakes during pregnancy on the subsequent development of appetite and food choices of her offspring has also been investigated. Maternal glucose concentrations are influenced by her total energy intake and by the proportions of carbohydrate, fat, and protein in her diet. A recent study investigated the association of maternal macronutrient and energy intake during pregnancy with the macronutrient and energy intake of her offspring [25]. Maternal dietary intakes of protein, fat, and carbohydrate in pregnancy were positively associated with the dietary intake of the same nutrients in both male and female offspring, and these associations were greater than those observed for paternal dietary intakes [25]. Furthermore, associations of maternal prenatal-offspring intakes were stronger than those for maternal postnatal-offspring intakes for protein and fat [25]. Thus, this study supports the conclusion which has also been drawn from a range of experimental animal studies as cited below, that there may be *in utero* programming of offspring appetite by maternal intake during pregnancy in the human.

## 4. Maternal Overnutrition: Critical Periods for the Development of Postnatal Obesity

There is a need to understand the separate or interdependent contributions of maternal pre pregnancy BMI, gestational weight gain, glycaemic control, and maternal macronutrient intake on the longer-term metabolic outcomes for the offspring, including the metabolic responses to an obesogenic diet. This understanding would help inform the evidence base for effective nutritional interventions in women before and during pregnancy. It is clearly difficult in human populations to determine the impact of exposure to a high maternal BMI during the preconceptional period separately from exposure to a high maternal BMI during any subsequent stage of pregnancy on the metabolic outcomes for the offspring. Most women who enter pregnancy heavy remain so during pregnancy and are also at greater risk of development of glucose intolerance during late gestation. 

A range of experimental animal studies have provided insights into the mechanisms that may underpin the early programming of a life of obesity, but as highlighted below there are relatively few experimental studies which have addressed the impact of maternal dietary interventions imposed during different periods of pregnancy on the metabolic outcomes for the offspring.

## 5. Animal Models of Maternal Overnutrition

### 5.1. The Early Programming of Obesity in the Rodent

The effects of obesity on oocyte quality and early embryo development have been assessed by Minge and colleagues using a mouse model of diet-induced obesity [26]. Embryos isolated from either obese or control-mated females were cultured *in vitro* in order to monitor their development. Although a difference in fertilisation rates was not observed, embryos from obese females exhibited slower development to the four- to eight-cell stage and through to the blastocyst stage. It was also reported in this study that defects in oocyte developmental competence caused by obesity were reversed by treatment with insulin sensitisers before conception. Thus, maternal obesity and peripheral insulin sensitivity are important from as early as the preconceptional period in determining developmental outcomes. This is important in the context that most animal models of maternal overnutrition include exposure to an increase in energy intake and/or a diet which is high in fat and/or sugar including exposure to “junk food” style diets from before conception and throughout pregnancy [27–31]. 

There have been several excellent recent papers of the impact of maternal overnutrition in rodents on the postnatal metabolic phenotype of the pups and offspring in later life [32–34]. In most instances, it has been reported that maternal overnutrition in the rat leads to a consistent increase in body fat mass, poor glucose tolerance, insulin resistance, and an increase in appetite in the offspring [32–35]. These studies provide important information on the metabolic signalling pathways that are perturbed in skeletal muscle, liver, and in visceral and subcutaneous adipose tissue. Interestingly, however, a recent systematic paper of those animals models which have used exposure to maternal high-fat feeding throughout pregnancy identified that there was a paucity of data which characterised what aspects of the maternal metabolic state were related to the specific postnatal outcomes. The authors of this paper commented that this limited the capacity to draw links between the maternal phenotype and the metabolic outcomes in the offspring in these models [36]. Important outcomes from this systematic paper were firstly that all offspring born to obese mothers had perturbed glycaemic control in postnatal life and secondly that poor glycaemic control was also observed in offspring from nonobese, high-fat-fed mothers [36]. Importantly, there was not convincing evidence that there was a hyperphagic phenotype in offspring exposed to a maternal diet which was high in fat and low in carbohydrate [36]. In contrast, there is consistent evidence for a hyperphagic phenotype in offspring exposed to a maternal diet that is high in sugar including “junk food” and cafeteria-style diets [27–31]. This is consistent with a wide range of studies in the rodent that have reported that exposure of the fetal and neonatal brain to conditions in which there is hyperglycaemia, hyperinsulinaemia, and/or hyperleptinaemia can result in the programming of postnatal appetite [37]. 

One important study determined the impact of feeding nonpregnant rats with 15% excess calories/day for 3 weeks prior to mating such that dams entered pregnancy obese or lean [38]. After mating, the dams were all placed on the same diet for the remainder of pregnancy, and the pups were cross-fostered at birth to normal-weight mothers to ensure that the nutritional intervention was restricted to pregnancy. Offspring from the obese dams gained greater body weight and had a higher percentage body fat than the offspring from lean dams when fed a high-fat diet in postnatal life. While the nutritional intervention in this study was restricted to the 3-week period prior to conception, the dams which entered pregnancy obese remained obese throughout pregnancy, and the increase in offspring adiposity might therefore reflect the impact of overnutrition and obesity in both the periconceptional period and the remainder of of pregnancy. Another key study placed obese female rats on a control chow diet from one month before mating for the remainder of pregnancy and lactation and compared the outcomes with those from obese female rats maintained on a high fat diet from before and during pregnancy and throughout lactation [39]. At 21 days after birth, serum triglycerides, leptin and insulin were increased in the offspring from the obese mother on a high fat diet, but not the offspring of the obese mother fed on the control diet. An increase in body fat mass in the offspring of high fat fed mothers at 5 months of age was partially “reversed” in offspring of the chow fed, previously obese mothers [39]. This study demonstrates the effectiveness of a weight loss intervention which extends from before and throughout pregnancy. It is not possible, however, to conclude whether the impact of the dietary intervention on the previously obese rat was a consequence of the decrease in her pre pregnancy weight and fat mass, gestational weight gain, or exposure to a high-fat diet during lactation.

### 5.2. The Early Programming of Obesity in the Sheep

There have been a range of studies using the sheep as a large animal model of pregnancy on the impact of exposure to maternal obesity on the fetal and postnatal lamb. The sheep is an excellent model for the study of the early development of later obesity. In the sheep, as in the human, the development of adipose tissue and of the hypothalamic neural network which regulates appetite and energy balance in later life occur before birth. This is unlike the rodent, where fat development and the appetite regulatory system in the hypothalamus each develop after birth [37, 40]. In an important series of studies, it has been reported that exposure of ewes to a diet containing 150% metabolic requirements from 60 days before conception and extending throughout pregnancy resulted in changes in adipose tissue development, glucose tolerance, and appetite regulation in the adult offspring [41]. In these studies, the adult offspring from obese and control-fed mothers were exposed to a 3-month “feeding challenge.” During the feeding challenge, offspring from obese ewes consumed more food and at the end of the challenge had a higher percentage of body fat than offspring from control ewes. There was also a decrease in insulin sensitivity in the offspring from obese ewes compared with their control counterparts [41]. In this study, body fat mass was measured using dual X-ray absorptiometry, and so it was not possible to determine whether the increase in body fat mass after the feeding challenge was a result of an increase in the visceral or subcutaneous fat depots. 

We have developed a model of maternal overnutrition in which pregnant ewes were overfed for the last month of pregnancy to determine whether exposure of the fetus to increased maternal glucose concentrations during late pregnancy would result in an increase in postnatal adiposity [42–44]. We found that maternal overnutrition imposed in this period resulted in an increase in fetal glucose and insulin concentrations and an upregulation of key adipogenic and lipogenic genes, including peroxisome-proliferator activator receptor *γ* (PPAR *γ*), leptin, and adiponectin within the perirenal fat depot of the late gestation sheep fetus [44]. We also found that there was an increased mass of subcutaneous, but not visceral fat present in the male and female lambs of these overnourished ewes at one month of age [42]. Furthermore, in the lambs of overnourished ewes, there was a decrease in the hypothalamic expression of the leptin receptor with increasing body fat mass and a loss of the positive relationship between the expression of a hypothalamic appetite inhibitory peptide (cocaine amphetamine-regulated transcript) and body fat mass [42].

Thus, it appears that exposure to maternal and fetal hyperglycaemia in late pregnancy alone can result in an increase in adiposity and in changes in the hypothalamic neural network which would limit the appropriate response to an increase in body fat mass and energy intake in the postnatal animal.

Relatively few animal studies have attempted to determine the impact of maternal obesity restricted to the “periconceptional period” alone on the development of adiposity in the offspring. The periconceptional period is an important period for intervention because as highlighted earlier there appears to be an association between maternal pre pregnancy BMI and an increased body fat mass in the offspring. Furthermore, dietary intervention in overweight or obese women is relatively more feasible in the pre- or periconceptional period.

#### 5.2.1. Definition of What Constitutes the “Periconceptional Period”

The periconceptional period includes the period extending from oocyte maturation to early gestation. In a number of studies in the sheep, nutritional interventions are imposed during a periconceptional period which extends from around 60 days before to 30 days after conception which covers the period of oocyte maturation, implantation, and placentation [45, 46]. Nutritional interventions which extend into the placentation period may affect placental growth and the placental transfer efficiency of substrates to the fetus during early or late pregnancy [47]. We have proposed therefore that the term “periconceptional” should be used to refer to the developmental stages which include some or all of the following early events: oocyte maturation, follicular development, conception, and embryo/blastocyst growth up until implantation [48]. When maternal nutritional interventions extend beyond implantation to include early placentation, then it may be more appropriate to describe these interventions as occurring during “early gestation.”

#### 5.2.2. Exposure to Maternal Overnutrition in the Periconceptional Period and Programming of Later Obesity in the Sheep

**Figure 1:**
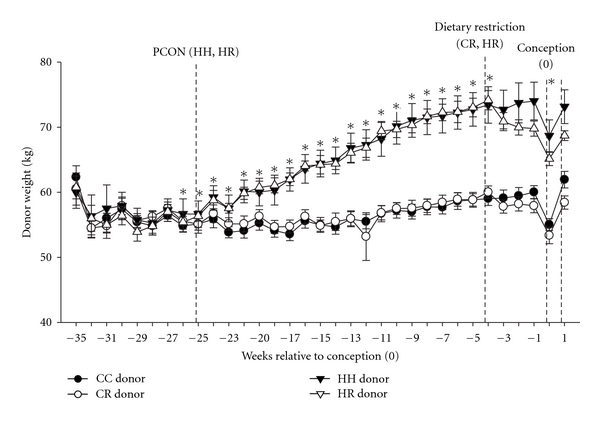
Weight of donor ewes during the nutritional feeding protocol from 35 weeks before conception to 1 week after conception (CC: closed circle; CR: open circle; HH: closed triangle; HR: open triangle). *denotes a significant difference between the weight of the donor ewes in the HH and HR groups compared to the CC and CR groups (*P* < 0.05) (reprinted from Rattanatray et al., 2010 [49]).

We have developed a model in which nonpregnant ewes were either overnourished or normally nourished for at least 4 months before artificial insemination [49, 50]. In two subgroups of non pregnant ewes, a 4-week period of dietary restriction was imposed before and after artificial insemination to result in 4 periconceptional treatment groups: periconceptional overnutrition with (HR) or without (HH) dietary restriction and control nutrition with (CR) or without (CC) dietary restriction [49]. Around a week after conception, single embryos were transferred from these donor ewes to nonobese recipient ewes which were then maintained on a control diet for the remainder of pregnancy. The ewes which were overnourished during the periconceptional period were heavier than the control ewes during this period (Figure 1). Recipient nonobese ewes were maintained at a normal body condition score from the start of the donor ewe feeding regime to the time of conception. This model therefore isolates the effect of exposure to maternal obesity during the periconceptional period alone.

**Figure 2:**
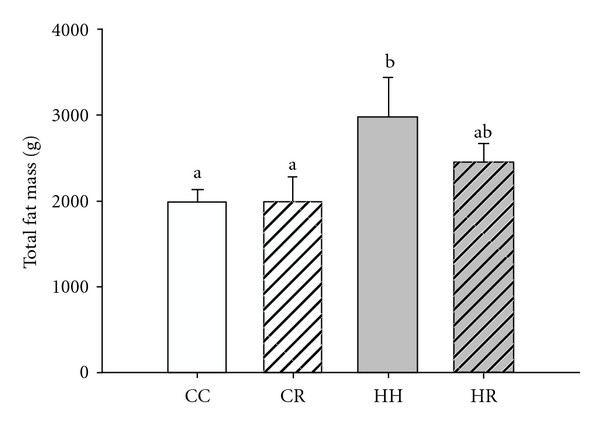
Effect of periconceptional overnutrition and/or dietary restriction on the total fat mass of female lambs at 4 months of age (CC: open bar; CR: striped bar; HH: grey bar; HR: grey striped bar). Different superscripts (e.g., a, b) denote a significant difference of total fat mass between the treatment groups (*P* < 0.05) (reprinted from Rattanatray et al. 2010 [49]).

We found that there was a gender-specific effect of periconceptional overnutrition on the body fat mass of the 4-month-old lambs. Periconceptional overnutrition resulted in an increase in total fat mass in female, but not male lambs [49]. Interestingly, maternal dietary restriction imposed in the overnourished ewe during the periconceptional period ablated the development of an increase in total body fat in the female lambs (Figure 2). There was also a significant relationship between the total fat mass of female lambs at 4 months of age and the weight of the donor ewe at conception. The greatest impact of periconceptional overnutrition was on the perirenal and omental fat depots in the female lamb and the weights of these depots were also higher in female than male lambs in all nutritional groups. The greater impact of periconceptional overnutrition in the female lamb, may be related to the higher level of expression of adipogenic and lipogenic genes, for example, G3PDH and lipoprotein lipase (LPL) present in adipose tissue in female, compared to male lambs.

Exposure of the oocyte and early embryo to a high plane of maternal nutrition therefore resulted in a greater postnatal capacity to synthesise and store triglycerides in female lambs. We have speculated that the early embryo may respond to maternal overnutrition to program changes within metabolic pathways which ultimately result in a more efficient deposition of fat in visceral fat depots in postnatal life. It may be that this early response is on the basis that the postnatal nutritional environment will match the high nutritional environment experienced by the embryo and that an increased capacity to store fat will be required in postnatal life.

Interestingly we found that there was no effect of periconceptional overnutrition on the level of expression of PPAR *γ*, leptin, and adiponectin in the perirenal, omental, or subcutaneous fat depots in lambs at 4 months of age [49]. Thus, exposure of the sheep embryo to periconceptional overnutrition results in an increased visceral adiposity with no increase in adipose PPAR *γ* or leptin expression, whereas exposure to maternal overnutrition in late gestation results in an increase in PPAR *γ* and leptin expression in fetal visceral fat and an increase in subcutaneous fat mass in postnatal life. These differences may reflect differences in timing between the study endpoints (4- versus 1-month postnatal age, resp.), or it may be that different signalling systems are activated in adipocytes after exposure to maternal overnutrition during the periconceptional and late gestational periods. Periconceptional overnutrition may result in epigenetic changes induced in the germ layers of the embryo associated with an increase in the differentiation, proliferation, and/or hypertrophy of visceral adipocytes.

Thus, the early programming of later obesity may result from “two hits,” the first occurring as a result of maternal overnutrition during the periconceptional period and the second occurring as a result of increased fetal nutrition in late pregnancy [51].

#### 5.2.3. Dietary Intervention during the Periconceptional Period: Metabolic Benefits

As noted above, we found that placing the obese ewe on a dietary regime where the energy intake was reduced to 70% of normal for one month before and one week after conception only resulted in an ablation of the effects of maternal overnutrition on the total fat mass in the female lamb [49]. This is an important finding as it highlights that nutritional intervention in the periconceptional period can ablate the effects of a high maternal pre pregnancy weight on offspring adiposity. One issue, however, is whether such a dietary restriction regime has any other positive or negative metabolic or endocrine consequences for the offspring.

#### 5.2.4. Dietary Intervention during the Periconceptional Period: Metabolic and Endocrine “Costs”

It has previously been shown that a severe nutritional restriction imposed in ewes with a body weight in the normal range across both the periconceptional and early gestation periods (from 60 days before until 30 days after mating) resulted in a decreased glucose tolerance in the 10-month-old offspring [45]. In this latter study, this period of undernutrition resulted in an increase in the glucose and insulin areas under the curve in response to a glucose tolerance test at 4 and at 10 months postnatal age [45]. It is not known, however, whether a similar period of dietary restriction in obese ewes would have similar metabolic consequences in the offspring. It has also been demonstrated that moderate dietary restriction imposed during the periconceptional period results in an increase in fetal arterial blood pressure and in an earlier activation of the fetal prepartum cortisol surge [52, 53].

**Figure 3:**
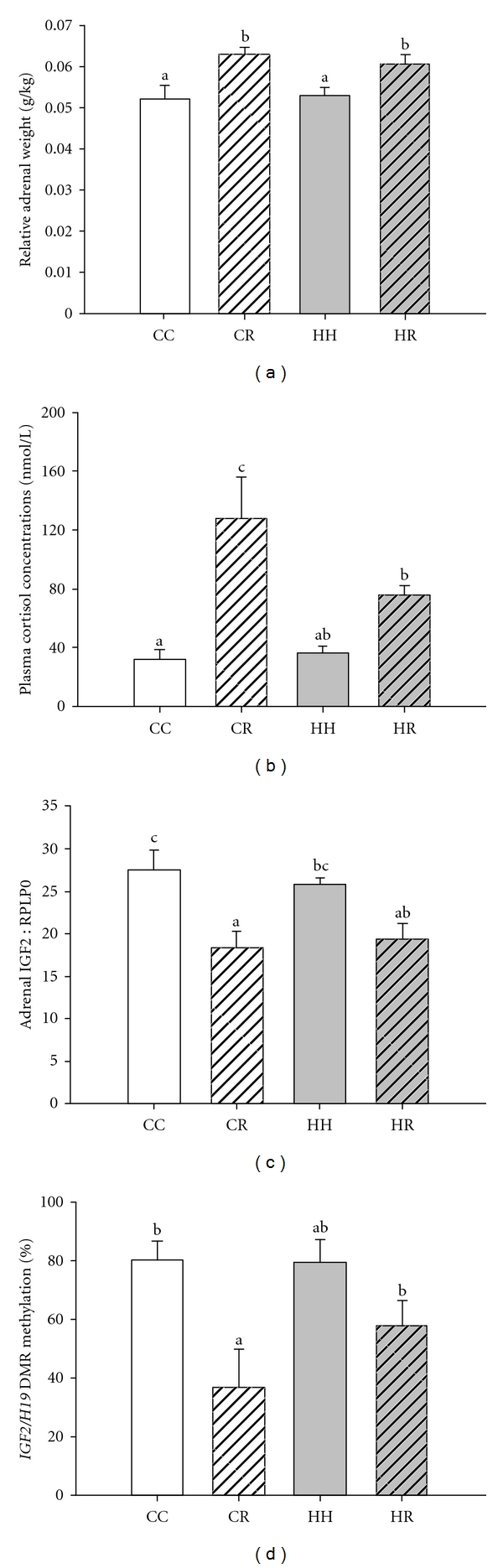
Relative adrenal weight (a) in the CC, CR, HH, and HR lambs (CC: open bar; CR: striped bar; HH: grey bar; HR: grey striped bar), plasma cortisol concentrations (b) in response to stress in female lambs at postnatal week 12, adrenal IGF2 mRNA expression (c), and IGF2/H19 DMR methylation levels (d) in lambs at postnatal week 16 (from Zhang et al., 2010 [50]). Different superscripts a, b, and c denote treatment groups which are significantly different from each other (*P* < 0.05).

We have recently determined whether exposure to a moderate restriction of energy intake in obese and normal weight ewes results in changes in the development of the hypothalamo-pituitary-adrenal axis and the stress response in the offspring. We have found that dietary restriction imposed during the periconceptional period in either normal weight or obese ewes resulted in an enhanced cortisol response to stress in female lambs at 3-4 months of age (Figure 3) [50]. In this study, the adrenal gland was also bigger at 4 months of age in male and female lambs which were conceived in either normal weight or obese ewes which had been exposed to dietary restriction during the periconceptional period. The increase in adrenal growth was associated with a decrease in the adrenal expression of the insulin-like growth factor 2 (IGF 2). IGF2 is expressed in a parent-of-origin-specific manner from the paternal gene and has been implicated in the regulation of adrenal growth and steroidogenesis in the fetal sheep [54]. Epigenetic modifications play a vital role in the transmission of the parental identity of particular alleles through the germline; this is achieved through complex mechanisms typified by cytosine methylation of “imprinting control regions” (ICR). For *IGF2*, the ICR resides within a differentially methylated region (DMR) 4 kb upstream of the neighbouring nonprotein coding *H19* gene. When the DMR is methylated, *IGF2* is expressed, and conversely when the DMR is unmethylated, *IGF2* expression is repressed by *H19 *[55]. Using combined bisulphite restriction analysis (COBRA), we found that the lamb adrenals in the CR group carried significantly less methylation in the *IGF2/H19* DMR when compared to the CC and HH groups, and this effect was present in both male and female lambs [50]. Bisulphite sequencing of a subset of animals from each group revealed that the loss of methylation observed in the CR group was marked, with most animals exhibiting a complete loss of methylation. It was also of note that some animals in the HR group exhibited loss of *IGF2/H19* DMR methylation. Thus, the increase in adrenal weight in the CR and HR lambs was paradoxically associated with a decrease in the expression of an adrenal growth factor which in turn was associated with decreased level of methylation in the proximal CTCF binding site in the DMR region of the *IGF2/H19* gene in these groups. There was no change, however, in the methylation status of *IGF2R* or in IGF2R mRNA expression in the adrenals of the lambs in either the CR or HR groups. It remains to be determined whether the decrease in adrenal IGF2 mRNA expression is a consequence of the epigenetic changes in adrenal *IGF2 *or whether there are other factors driving adrenal growth in these animals which in turn suppress IGF2 gene expression within the adrenal. It has recently been reported that people whose mothers were exposed to famine during the Dutch Hunger Winter in 1944-1945 had less *IGF2* methylation in blood cells in adult life compared with their unexposed same-sex siblings [56]. In particular, epigenetic changes were found among individuals who had been exposed to famine in early gestation and who had a normal birth weight. In contrast, exposure to famine in late gestation was associated with a low birth weight, but not with epigenetic changes in the blood cells in later life [56].

Thus, studies on the effects of maternal undernutrition imposed during the periconceptional period highlight the need to be cautious about the level and length of any dietary intervention regime imposed around the time of conception as not all of the metabolic and endocrine effects of dietary restriction in this period may be beneficial in the longer term. 

Thus, there appear to be a series of changes including epigenetic modifications in a number of key genes induced in the embryo after exposure to either maternal over- or undernutrition. These may result in metabolic and endocrine changes which result in responses consistent with the expectation of either a “life of adversity” or of a “life of plenty” with high levels of postnatal nutrition. Critically, there is evidence that metabolic and endocrine changes may be induced in offspring after imposition of dietary restriction during the pre pregnancy period in either normal weight or obese mothers. This is important in the context of the dietary advice given to overweight or obese women who are seeking to become pregnant as it is critical that any dietary intervention imposed during the periconceptional period is evidence based and does not incur a further metabolic or endocrine cost in the offspring.

## 6. Summary

**Figure 4:**
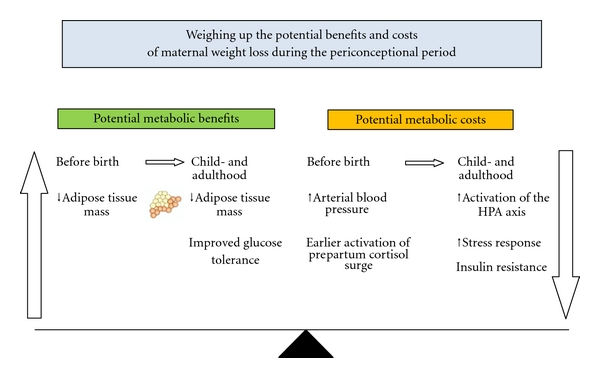
Diagram summarising the potential benefits and costs of maternal weight loss during the periconceptional period.

It is clear that further work is required to determine the separate or interdependent contributions of maternal pre pregnancy BMI and gestational weight gain, glycaemic control, and macronutrient intake during pregnancy on the metabolic outcomes in the offspring. It appears from recent studies in the sheep that the early programming of later obesity may result from “two hits” related to the separate influences of maternal obesity experienced during the periconceptional period and during late gestation. Each of these exposures may act through different mechanisms to alter the propensity for triglyceride storage in specific fat depots after birth. While a period of dietary restriction in overweight mothers may ablate the impact of maternal BMI on the programming of postnatal obesity, it is associated with an activation of the stress axis and a potential impact on glucose tolerance in the offspring (Figure 4). Thus, a high maternal pre pregnancy BMI may result in epigenetic changes within the embryo which predict the need for efficient fat storage in postnatal life. In contrast, weight loss in mothers with either a high or normal BMI may lead to epigenetic changes within the stress axis which predict the likely need to respond to adversity in later life. Thus, it is important to ensure that any dietary restriction interventions recommended for overweight or obese mothers are evidence based to allow an effective weighing up of the potential metabolic benefits and costs (Figure 4) for the offspring.

## References

[1] Poston L, Harthoorn LF, Van Der Beek EM, on behalf of contributors to the ILSI Europe Workshop (2011). Obesity in pregnancy: implications for the mother and lifelong health of the child. A consensus statement. *Pediatric Research*.

[2] Kumanyika S, Obarzanek E, Stettler N (2008). Population-based prevention of obesity: the need for comprehensive promotion of healthful eating, physical activity, and energy balance: a scientific statement from American heart association council on epidemiology and prevention, interdisciplinary committee for prevention (formerly the expert panel on population and prevention science). *Circulation*.

[3] Australian Bureau of Statistics Australian Social Trends.

[4] Flegal K, Carroll M, Ogden C, Johnson C (2002). Prevalence and trends in obesity among US adults, 1999-2000. *Journal of the American Medical Association*.

[5] Ogden CL, Yanovski SZ, Carroll MD, Flegal KM (2007). The epidemiology of obesity. *Gastroenterology*.

[6] La Coursiere D, Bloebaum L, Duncan J, Varner M (2005). Population-based trends and correlates of maternal overweight and obesity, Utah 1991–2001. *American Journal of Obstetrics and Gynecology*.

[7] Athukorala C, Rumbold AR, Willson KJ, Crowther CA (2010). The risk of adverse pregnancy outcomes in women who are overweight or obese. *BioMed Central*.

[8] Callaway LK, Prins JB, Chang AM, McIntyre HD (2006). The prevalence and impact of overweight and obesity in an Australian obstetrics population. *Medical Journal of Australia*.

[9] Nohr EA, Vaeth M, Baker JL, Sorensen TIA, Olsen J, Rasmussen KM (2008). Combined associations of prepregnancy body mass index and gestational weight gain with the outcome of pregnancy. *American Journal of Clinical Nutrition*.

[10] Oken E, Kleinman KP, Belfort MB, Hammitt JK, Gillman MW (2009). Associations of gestational weight gain with short- and longer-term maternal and child health outcomes. *American Journal of Epidemiology*.

[11] Rasmussen K, Yaktine A (2009). *Weight Gain During Pregnancy: Reexamining the Guidelines*.

[12] Siega-Riz AM, Viswanathan M, Moos MK (2009). A systematic review of outcomes of maternal weight gain according to the institute of medicine recommendations: birthweight, fetal growth, and postpartum weight retention. *American Journal of Obstetrics and Gynecology*.

[13] Curhan G, Willett W, Rimm E, Spiegelman D, Ascherio A, Stampfer M (1996). Birth weight and adult hypertension, diabetes mellitus, and obesity in US men. *Circulation*.

[14] Oken E, Taveras E, Kleinman K, Rich-Edwards J, Gillman M (2007). Gestational weight gain and child adiposity at age 3 years. *American Journal of Obstetrics and Gynecology*.

[15] Parsons TJ, Power C, Manor O (2001). Fetal and early life growth and body mass index from birth to early adulthood in 1958 British cohort: longitudinal. *British Medical Journal*.

[16] Whitaker RC (2004). Predicting preschooler obesity at birth: the role of maternal obesity in early pregnancy. *Pediatrics*.

[17] Rugholm S, Baker JL, Olsen LW, Schack-Nielsen L, Bua J, Sorensen TIA (2005). Stability of the association between birth weight and childhood overweight during the development of the obesity epidemic. *Obesity Research*.

[18] Smith J, Cianflone K, Biron S (2009). Effects of maternal surgical weight loss in mothers on intergenerational transmission of obesity. *Journal of Clinical Endocrinology and Metabolism*.

[19] Crowther CA, Hiller JE, Moss JR, McPhee AJ, Jeffries WS, Robinson JS (2005). Effect of treatment of gestational diabetes mellitus on pregnancy outcomes. *The New England Journal of Medicine*.

[20] Landon MB, Spong CY, Thom E (2009). A multicenter, randomized trial of treatment for mild gestational diabetes. *The New England Journal of Medicine*.

[21] Dabelea D, Hanson RL, Lindsay RS (2000). Intrauterine exposure to diabetes conveys risks for type 2 diabetes and obesity: a study of discordant sibships. *Diabetes*.

[22] Gillman MW, Oakey H, Baghurst PA, Volkmer RE, Robinson JS, Crowther CA (2010). Effect of treatment of gestational diabetes mellitus on obesity in the next generation. *Diabetes Care*.

[23] Catalano PM, Ehrenberg HM (2006). The short- and long-term implications of maternal obesity on the mother and her offspring. *British Journal of Obstetrics and Gynaecology*.

[24] Langer O, Yogev Y, Xenakis E, Brustman L (2005). Overweight and obese in gestational diabetes: the impact on pregnancy outcome. *American Journal of Obstetrics and Gynecology*.

[25] Brion MJ, Ness AR, Rogers I (2010). Maternal macronutrient and energy intakes in pregnancy and offspring intake at 10 y: exploring parental comparisons and prenatal effects. *American Journal of Clinical Nutrition*.

[26] Minge C, Bennett B, Norman R, Robker R (2008). Peroxisome proliferator-activated receptor-*γ* agonist rosiglitazone reverses the adverse effects of diet-induced obesity on oocyte quality. *Endocrinology*.

[27] Nivoit P, Morens C, Van Assche FA (2009). Established diet-induced obesity in female rats leads to offspring hyperphagia, adiposity and insulin resistance. *Diabetologia*.

[28] Bayol SA, Farrington SJ, Stickland NC (2007). A maternal ’junk food’ diet in pregnancy and lactation promotes an exacerbated taste for ’junk food’ and a greater propensity for obesity in rat offspring. *British Journal of Nutrition*.

[29] Morris MJ, Chen H (2009). Established maternal obesity in the rat reprograms hypothalamic appetite regulators and leptin signaling at birth. *International Journal of Obesity*.

[30] Rajia S, Chen H, Morris MJ (2010). Maternal overnutrition impacts offspring adiposity and brain appetite markers-modulation by postweaning diet. *Journal of Neuroendocrinology*.

[31] Kirk SL, Samuelsson AM, Argenton M (2009). Maternal obesity induced by diet in rats permanently influences central processes regulating food intake in offspring. *Plos One*.

[32] Rooney K, Ozanne SE (2011). Maternal over-nutrition and offspring obesity predisposition: targets for preventative interventions. *International Journal of Obesity*.

[33] Armitage JA, Taylor PD, Poston L (2005). Experimental models of developmental programming: consequences of exposure to an energy rich diet during development. *Journal of Physiology*.

[34] Armitage JA, Poston L, Taylor PD (2008). Developmental origins of obesity and the metabolic syndrome: the role of maternal obesity. *Frontiers of Hormone Research*.

[35] Armitage JA, Khan IY, Taylor PD, Nathanielsz PW, Poston L (2004). Developmental programming of the metabolic syndrome by maternal nutritional imbalance: how strong is the evidence from experimental models in mammals?. *Journal of Physiology*.

[36] Ainge H, Thompson C, Ozanne SE, Rooney KB (2010). A systematic review on animal models of maternal high fat feeding and offspring glycaemic control. *International Journal of Obesity*.

[37] McMillen IC, Robinson JS (2005). Developmental origins of the metabolic syndrome: prediction, plasticity, and programming. *Physiological Reviews*.

[38] Shankar K, Harrell A, Liu X, Gilchrist JM, Ronis MJJ, Badger TM (2008). Maternal obesity at conception programs obesity in the offspring. *American Journal of Physiology*.

[39] Zambrano E, Martinez-Samayoa PM, Rodriguez-Gonzalez GL, Nathanielsz PW (2010). Dietary intervention prior to pregnancy reverses metabolic programming in male offspring of obese rats. *Journal of Physiology*.

[40] McMillen IC, Rattanatray L, Duffield JA (2009). The early origins of later obesity: pathways and mechanisms. *Advances in Experimental Medicine and Biology*.

[41] Long NM, George LA, Uthlaut AB (2010). Maternal obesity and increased nutrient intake before and during gestation in the ewe results in altered growth, adiposity, and glucose tolerance in adult offspring. *Journal of Animal Science*.

[42] Muhlhausler BS, Adam CL, Findlay PA, Duffield JA, McMillen IC (2006). Increased maternal nutrition alters development of the appetite-regulating network in the brain. *Journal of the Federation of American Societies for Experimental Biology*.

[43] Muhlhausler BS, Roberts CT, Yuen BSJ (2003). Determinants of fetal leptin synthesis, fat mass, and circulating leptin concentrations in well-nourished ewes in late pregnancy. *Endocrinology*.

[44] Muhlhausler BS, Duffield JA, McMillen IC (2007). Increased maternal nutrition stimulates peroxisome proliferator activated receptor-*γ*, adiponectin, and leptin messenger ribonucleic acid expression in adipose tissue before birth. *Endocrinology*.

[45] Todd S, Oliver M, Jaquiery A, Bloomfield F, Harding J (2009). Periconceptional undernutrition of ewes impairs glucose tolerance in their adult offspring. *Pediatric Research*.

[46] Bloomfield FH, Oliver MH, Hawkins P (2003). A periconceptional nutritional origin for noninfectious preterm birth. *Science*.

[47] MacLaughlin SM, Walker SK, Roberts CT, Kleemann DO, McMillen IC (2005). Periconceptional nutrition and the relationship between maternal body weight changes in the periconceptional period and feto-placental growth in the sheep. *Journal of Physiology*.

[48] MacLaughlin SM, Muhlhausler BS, Gentili S, McMillen IC (2006). When in gestation do nutritional alterations exert their effects? A focus on the early origins of adult disease. *Current Opinion in Endocrinology and Diabetes*.

[49] Rattanatray L, MacLaughlin SM, Kleemann DO, Walker SK, Muhlhausler BS, McMillen IC (2010). Impact of maternal periconceptional overnutrition on fat mass and expression of adipogenic and lipogenic genes in visceral and subcutaneous fat depots in the postnatal lamb. *Endocrinology*.

[50] Zhang S, Rattanatray L, MacLaughlin SM (2010). Periconceptional undernutrition in normal and overweight ewes leads to increased adrenal growth and epigenetic changes in adrenal IGF2/H19 gene in offspring. *Journal of the Federation of American Societies for Experimental Biology*.

[51] Zhang S, Rattanatray L, McMillen IC, Suter CM, Morrison JL (2011). Periconceptional nutrition and the early programming of a life of obesity or adversity. *Progress in Biophysics and Molecular Biology*.

[52] Edwards LJ, McMillen IC (2002). Periconceptional nutrition programs development of the cardiovascular system in the fetal sheep. *American Journal of Physiology*.

[53] Edwards LJ, McMillen IC (2002). Impact of maternal undernutrition during the periconceptional period, fetal number, and fetal sex on the development of the hypothalamo-pituitary adrenal axis in sheep during late gestation. *Biology of Reproduction*.

[54] Ross JT, McMillen IC, Lok F, Thiel AG, Owens JA, Coulter CL (2007 ). Intrafetal insulin-like growth factor-I infusion stimulates adrenal growth but not steroidogenesis in the sheep fetus during late gestation. *Endocrinology*.

[55] Wood AJ, Oakey RJ (2006). Genomic imprinting in mammals: emerging themes and established theories. *Plos Genetics*.

[56] Heijmans BT, Tobi EW, Stein AD (2008). Persistent epigenetic differences associated with prenatal exposure to famine in humans. *Proceedings of the National Academy of Sciences of the United States of America*.

